# Co-Expression of Runx1, Hoxa9, Hlf, and Hoxa7 Confers Multi-Lineage Potential on Hematopoietic Progenitors Derived From Pluripotent Stem Cells

**DOI:** 10.3389/fcell.2022.859769

**Published:** 2022-04-28

**Authors:** Bo Yu, Bingyan Wu, Pingshan Hong, Huan Peng, Mengyun Zhang, Qi Zhang, Lijuan Liu, Xiaofei Liu, Yang Geng, Jinyong Wang, Yu Lan

**Affiliations:** ^1^ Key Laboratory for Regenerative Medicine of Ministry of Education, Institute of Hematology, School of Medicine, Jinan University, Guangzhou, China; ^2^ CAS Key Laboratory of Regenerative Biology, Guangzhou Institutes of Biomedicine and Health, Chinese Academy of Sciences, Guangzhou, China; ^3^ University of Chinese Academy of Sciences, Beijing, China; ^4^ GMU-GIBH Joint School of Life Sciences, Guangzhou Medical University, Guangzhou, China

**Keywords:** Runx1, Hoxa9, Hlf, Hoxa7, pluripotent stem cells, transcription factors, multi-lineage hematopoiesis

## Abstract

The intrinsic factors that determine the fundamental traits of engraftment ability and multi-lineage potential of hematopoietic stem cells (HSCs) remain elusive. The induction of bona fade HSCs from pluripotent stem cells (PSCs) in dishes is urgently demanded but remains a great challenge in translational medicine. *Runx1*, *Hoxa9*, *Hlf*, and *Hoxa7* are developmentally co-expressed during endothelial-to-hematopoietic transition and adult haematopoiesis. However, the expression of these factors fails to be turned on during *in vitro* hematopoietic induction from PSCs. Here, we established an inducible gene over-expression embryonic stem cell (ESC) line in which exogenous *Runx1*, *Hoxa9*, *Hlf*, and *Hoxa7* genes were tandemly knocked in. A population of induced hematopoietic progenitor cells (iHPCs) expressing Kit and Sca1 surface markers were successfully obtained *in vitro* from the gene edited-ESC line. Upon transplantation of the *Runx1-Hoxa9-Hlf-Hoxa7* ESC-derived iHPCs into irradiated immunodeficient mice, they can dominantly contribute to B cells, low proportions of T cells and myeloid cells. However, *Runx1*-*Hoxa9*-*Hlf* ESC-derived iHPCs only produced B lineage cells with extremely low contributions. Our study unveils that the coordination of *Runx1*, *Hoxa9*, *Hlf*, and *Hoxa7* led to generation of the hematopoietic progenitors with the capacity of multi-lineage hematopoietic reconstitution in the immunodeficient recipient mice.

## 1 Introduction

Hematopoietic stem cells (HSCs) emerge during embryogenesis and give rise to the adult hematopoietic and immune system, with the ability to self-renew and differentiate into lymphoid, myeloid, erythroid and other blood lineages (Chotinantakul and Leeanansaksiri, 2012; Laurenti and Gottgens, 2018). The hematopoietic system plays a fundamental role in regulating homeostasis and immune function. At present, hematopoietic regeneration is mainly achieved through allogeneic and autologous HSC transplantation in the treatment of blood-related diseases (Rogers and Casper, 2004). Despite wide clinical uses, the lack of timely HLA-matched donors and the tendency of allograft to lead to graft-versus-host disease hindering the application of allogeneic transplantation in clinical applications (Jacobsohn and Vogelsang, 2007). In spite of advances in HLA-typing to identify histocompatible donors, HLA-haploidentical stem cell transplantation somewhat relieves the pressure of bone marrow deficiency, graft-versus-host disease remains a significant cause of morbidity and mortality in patients receiving unrelated donor transplants (Petersdorf, 2013; Mallhi et al., 2020). Thus, *de novo* generation of isogenic HSCs from patient-derived cells has been a long-standing goal of stem cell biologists working in the field of developmental hematopoiesis.

In a previous study, we have identified eight candidate transcription factors that were previously considered essential for hematopoiesis but failed to be expressed during hematopoietic differentiation from pluripotent stem cells (PSCs) *in vitro* (Guo et al., 2020), including *Hoxa5* (Sugimura et al., 2017), *Hoxa7* (So et al., 2004), *Hoxa9* (Ramos-Mejia et al., 2014), *Hoxa10* (Magnusson et al., 2007), *Hlf (* *Komorowska et al., 2017* *)*, *Ikzf1* (Davis, 2011), Nkx2-3 (Nagel et al., 2017), and *Setbp1* (Oakley et al., 2012). We have reported that the synergistic action of transcription factors *Runx1* and *Hoxa9* led to functional T-lymphocyte regeneration *in vivo*, but did not achieve long-term multi-lineage hematopoiesis (Guo et al., 2020). In this study, we further used the “i*Runx1+Hoxa9*+Xi” tandem-factor-knockin strategy to explore whether the introduction of HSC-specific transcription factors might endow PSC-derived hematopoietic endothelium with the potential to implant multi-lineage hematopoiesis *in vivo*. Here, we show that simultaneous expression of *Runx1*, *Hoxa9*, *Hlf*, and *Hoxa7* drives embryonic stem cells (ESCs) differentiation towards engraftable induced hematopoietic progenitor cells (iHPCs), which gave rise to T cells, B cells, and myeloid cells in transplanted mice.

## 2 Material and Methods

### 2.1 Mice

B-NDG (NOD-*Prkdc* ^*Scid*^ *IL2rg* ^*tm1*^/Bcgen, CD45.1^+^) mice were purchased from Biocytogen Jiangsu Co., Ltd (Jiangsu, China). Mice were maintained in individually ventilated racks in the SPF-grade animal room of the Guangzhou Institutes of Biomedicine and Health, Chinese Academy of Sciences (GIBH, CAS, China) and given sterilized food.

### 2.2 Cell Line Constructs

To generate green fluorescent protein positive ESCs (GFP-ESCs), the *CAG Pr-GFP-PGK Pr-PuroR* cassette was inserted into the *Hipp11* locus of mouse ESCs (C57BL/6 background, CD45.2^+^) by CRISPR/Cas9-mediated gene knockin system. The target clones (GFP-ESCs) were selected by puromycine (1 μg/ml, Thermo Fischer Scientific), and the expression of GFP was confirmed by microscopy analysis and flow cytometry. In our study, p2a derived from porcine teschovirus-1 was used between *Runx1* and *Hoxa9* and between *Hoxa9* and *Hlf*, and e2a derived from equine rhinitis A virus was used between *Hlf* and *Hoxa7*. The genes of *Runx1*, *Hoxa9*, *Hlf*, and *Hoxa7* following CAG promoter were directionally cloned into the expression vector based on ClonExpress® technology (Vazyme) to form a gene tandem co-expression cassette: *Runx1*-*p2a*-*Hoxa9*-*p2a*-*Hlf*-*e2a*-*Hoxa7*. Simultaneously, the *rtTA*, *TRE*, and hygromycin (Hygro B) resistance selection marker sequences were also integrated to form a complete expression vector with hygromycin (Hygro B) resistance selection function and the genes of *Runx1*, *Hoxa9*, *Hlf*, and *Hoxa7* we inserted being inducible by doxycycline. Then the *CAG Pr-rtTA-3 × Stop-TRE-Runx1-p2a-Hoxa9-p2a-Hlf-e2a-Hoxa7-PGK Pr-HygroR* cassette we have constructed was knocked into the *Rosa26* locus of GFP-ESCs by cre-loxp-specific homologous recombination to generate i*Runx1*-*Hoxa9*-*Hlf*-*Hoxa7* (iR9F7) ESCs. Similarly, a *CAG Pr-rtTA-3 × Stop-TRE-Runx1-p2a-Hoxa9-p2a-Hlf-PGK Pr-HygroR* cassette was inserted into the *Rosa26* locus of GFP-ESCs by homologous recombination to generate *iRunx1-Hoxa9-Hlf* (iR9F) ESCs, and *a CAG Pr-rtTA-3 × Stop-TRE-Runx1-p2a-Hoxa9-e2a-Hoxa7-PGK Pr-HygroR* cassette was inserted into the *Rosa26* locus of GFP-ESCs to generate *iRunx1-Hoxa9-Hoxa7* (iR9A7) ESCs. The target clones (iR9F7-ESCs, iR9F-ESCs, and iR9A7-ESCs) selected by hygromycin B (150 μg/ml, Invivogen) were further cultured in ES medium containing doxycycline (1 μg/ml, Sigma). The induced expression of *Runx1*, *Hoxa9*, *Hlf,* and *Hoxa7* of iR9F7-ESCs were confirmed by qPCR.

### 2.3 Cell Culture

Mouse embryonic fibroblasts (MEFs) were derived from embryonic day 13.5 C57BL/6 mouse embryos. MEFs were incubated in DMEM/high glucose (Hyclone) with 10% fetal bovine serum (FBS) (Natocor) supplemented with 1% nonessential amino acids (NEAA, Gibco). C57BL/6 mouse embryonic stem cells (Biocytogen) were seeded into 12-well plate containing feeder layers in ES medium with DMEM/high glucose, 15% FBS (Gibco), 1% NEAA, 1% GlutaMAX (Gibco), 1% Sodium Pyruvate (Gibco), 0.1 mM β-mercaptoethanol (Gibco), 1 μM PD0325901 (Selleck), 3 μM Chir99021 (Selleck) and 1000 U/mL LIF. The OP9-DL1 cells (GFP^+^) were incubated in α-MEM (Gibco) supplemented with 20% FBS (CellMax). The AFT024 cell lines (ATCC) each with the gene encoding mIL3, mIL6, mSCF, and hFlt3L respectively been knocked in, were cultured in DMEM/high glucose supplemented with 10% FBS (Natocor), 0.1 mM β-mercaptoethanol, and 1% Sodium Pyruvate.

### 2.4 Hematopoietic Differentiation

Following the two-step method described in our previous study (Guo et al., 2020), mouse ESCs were detached by 0.05% Trypsin-EDTA (Gibco) and resuspended in the basic differentiation medium (BDM): IMDM, 15% FBS (Gibco), 200 μg/ml iron-saturated transferrin (Sigma), 0.1 mM β-mercaptoethanol (Sigma), 1% GlutaMAX, and 50 μg/ml ascorbic acid (Sigma). The digested ESCs and feeder cells were plated into the 0.1% gelatin-coated well for 30 min, then the floating cells but not the rapidly attached feeder cells were collected for the subsequent differentiation. For embryoid body (EB) generation, the ESCs were resuspended at 100,000 cells/mL in the BDM supplemented with 5 ng/ml BMP4 (Peprotech) and plated at 20 uL/drop for inverted culture in 15 cm dishes for 2.5 days, then replanted them into gelatinized plates in BDM supplemented with cytokines (5 ng/ml BMP4 and 5 ng/ml VEGF, Novoprotein) for 3.5 days. At day 6, the medium was changed to BDM supplemented with 2% conditioned medium derived from the supernatants of AFT024-mIL3, AFT024-mIL6, AFT024-hFlt3L, and AFT024-mSCF cell cultures. Doxycycline (1 μg/ml, Sigma) was added from day 6. The medium was replaced every other day. 2 × 10^4^ OP9-DL1 stromal cells were seeded into a 12-well plate 12 h prior to the hematopoietic maturation step in EM medium (α-MEM, 15% FBS (Hyclone), 200 μg/ml iron-saturated transferrin, 0.1 mM β-mercaptoethanol, 1% GlutaMAX, 50 μg/ml ascorbic acid, 2% conditioned medium derived from supernatants of AFT024-mIL3, AFT024-hFlt3L, and AFT024-mSCF cell culture and 1 μg/ml doxycycline. Then, 1,000–3,000 sorted iHECs were seeded into each well for hematopoietic maturation. The EM medium was half replaced every 2 days.

### 2.5 Transplantation of iHPCs

Eight-to-ten-week-old B-NDG mice were sub-lethally irradiated (2.25 Gy) by an X-ray irradiator (RS 2000, Rad Source Inc.). Three million ESC-derived iHPCs were injected into each irradiated B-NDG mouse *via* retro-orbital veins. Doxycycline (1 mg/ml) was maintained in the drinking water to continuously induce the expression of the inserted genes. We also sorted Lineage^−^Scal^+^Kit^+^ (LSK) cells from the bone marrow of the primary recipients 6 weeks post-transplantation and performed secondary transplantation using B-NDG mice as recipients, with 500 LSK cells per recipient mouse.

### 2.6 Flow Cytometry and Cell Sorting

Single-cell suspensions were prepared in phosphate-buffered saline (PBS) containing 2% FBS and filtered by 70 μm filter. After blocked by Fc (CD16/32) (Biolegend) antibody, cells were stained with combinations of fluorochrome-conjugated monoclonal antibodies, including Kit (2B8, eBioscience), CD31 (390, eBioscience), CD41 (eBioMWReg30, eBioscience), CD45 (30-F11, eBioscience), CD201 (eBio1560,eBioscience), CD45.2 (104, eBioscience), CD2 (RM2-5, eBioscience), CD3 (145-2C11, eBioscience), CD4 (GK1.5,eBioscience), CD8a (536.7, eBioscience), B220 (RA3-6B2, eBioscience), Mac1 (M1/70, BioLegend), NK1.1 (PK136, BioLegend), Ter119 (TER-119, eBioscience), Gr1 (RB6-8C5,eBioscience), IgM (II/41, eBioscience), IgD (11–26c.2a, BioLegend), Sca-1 (D7,eBioscience), CD19 (eBio1D3, eBioscience), CD43 (eBioR2/60, eBioscience), CD71 (R17217, eBioscience), CD61 (2C9.G2, BioLegend), CD127 (A7R34, BioLegend), CD135 (A2F10, BioLegend), Ly-6D (49-H4, eBioscience), CD48 (HM48-1, eBioscience), CD150 (TC15-12F12.2, BioLegend), Ly51 (6C3, BioLegend), CD24 (M1/69, BioLegend), Streptavidin Alexa Fluor® 700 (Invitrogen), and Streptavidin PE-Cy7 (BioLegend). DAPI solution (Sigma), or PI solution (BioLegend) was used to exclude dead cells. Cells were analyzed with Fortessa cytometer (BD Biosciences) and sorted with Arial II cytometer (BD Biosciences). The flow cytometry data were analyzed with FlowJo software (BD Biosciences).

### 2.7 Genomic PCR and RT-PCR

Genomic DNA was purified with the Discover-sc Single Cell WGA Kit (Vazyme) from the regenerated cells which were sorted from recipient spleens 16 weeks post-transplantation. DNA samples (200 ng of each) were amplified with a S1000^TM^ Thermal Cycler (BIO-RAD). In brief, we firstly used genomic DNA for the first round of PCR (low brightness), and then used the purified and recovered PCR product as a template for the second round of PCR, and eventually used this PCR products as a sample for agarose gel electrophoresis. Agarose gel electrophoresis was performed using 1.2% agarose gel, 1% TAE buffer. DL5000 DNA Marker (Tsingke) was used to indicate DNA molecular weight. PCR sequencing was performed using the primer pairs flanking the *Runx1*-p2a-*Hoxa9*-p2a-*Hlf*-e2a-*Hoxa7* element (Supplementary Figure S1). We used Trizol (Sigma) to lyse cells, then extracted RNA by isopropanol precipitation. ReverTra Ace qPCR RT Master Mix with gDNA Remover (FSQ-301, TOYOBO) kit was used for reverse transcription experiments. Hieff qPCR SYBR Green Master Mix was used to prepare the PCR reaction mixture before performing the reaction on the fluorescence quantitative PCR instrument (Bio-Rad, CFX-96). The primer information for genomic PCR, RT-PCR, and sequencing is provided in Supplementary Figure S1. PCR products were separated by agarose gel electrophoresis and were visualized by ethidium bromide staining. All PCR products shown correspond to expected molecular sizes.

## 3 Results

### 3.1 Construction of Inducible *Runx1*-*Hoxa9*-*Hlf*-*Hoxa7* ESC Line

**FIGURE 1:**
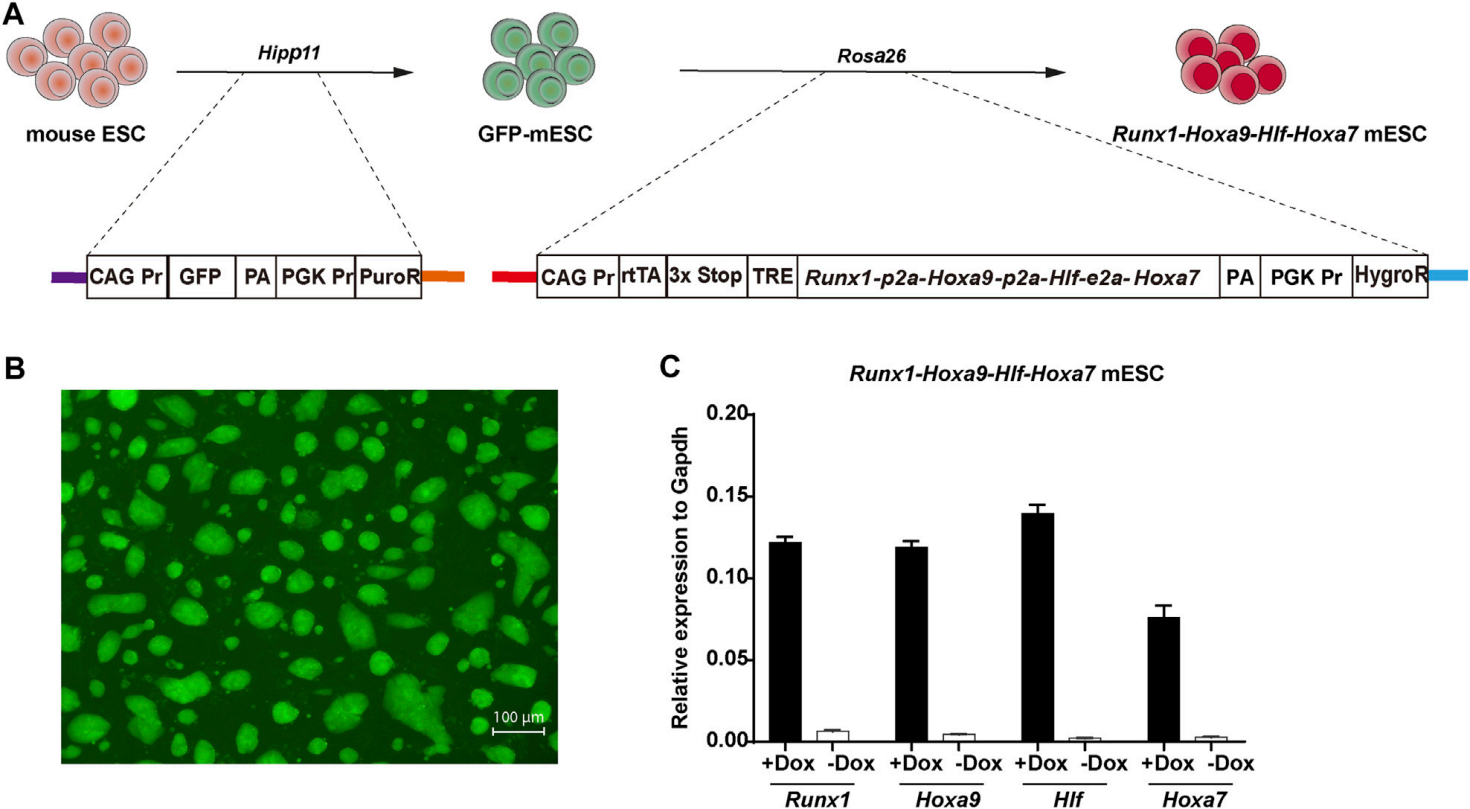
Construction of *Runx1-Hoxa9-Hlf-Hoxa7* mouse ESC line **(A)** Schematic overview of the i*Runx1-Hoxa9-Hlf-Hoxa7* (iR9F7) mouse ESC line construction. A *CAG Pr-GFP-PGK Pr-PuroR* DNA cassette was inserted into the *Hipp11* locus of CD45.2^+^ C57BL/6 mouse ESCs by using a CRISPR/Cas9-knock-in system to form GFP-ESC clones which were selected by puromycine (1 μg/ml). Next, a *rtTA-TRE-Runx1-Hoxa9-Hlf-Hoxa7-HygroR* DNA cassette was inserted into the *Rosa26* locus of GFP-ESCs by cre-loxp-specific homologous recombination to form iR9F7-ESC clones which were selected by hygromycin B (150 μg/ml) **(B)** Representative image showing the iR9F7-ESC clones observed by fluorescence microscopy **(C)** Quantitative real-time PCR analysis of gene expression of *Runx1*, *Hoxa9*, *Hlf*, and *Hoxa7* in iR9F7-ESCs.

We previously achieved regeneration of hematopoietic progenitors with dominant T lineage potential using a *Runx1-Hoxa9* knockin mouse ESC line (Guo et al., 2020). To evaluate the effects of *Hlf* and *Hoxa7* transcription factors on hematopoietic regeneration from PSCs *in vitro*, we construct a doxycycline inducible *Runx1*-*Hoxa9*-*Hlf*-*Hoxa7* knockin mouse ESC line (iR9F7-ESCs, C57BL/6 background) by inserting the *Runx1*-*Hoxa9*-*Hlf*-*Hoxa7* polycistronic into the *Rosa26* locus of mouse ESCs *via* homologous recombination. In addition, we added a sequence of constitutively expressing GFP fluorescent protein into the *Hipp11* locus of mouse ESCs (GFP-ESCs, C57BL/6 background) to monitor the progeny cells (Figure 1A). The GFP protein signals in ESCs were identified in the GFP-ESCs (Figure 1B). The addition of doxycycline successfully drives the conditional expression of exogenous *Runx1*, *Hoxa9*, *Hlf*, and *Hoxa7* (Figure 1C). Thus, we build a mouse ESC line for conditionally expressing the tandem *Runx1*, *Hoxa9*, *Hlf*, and *Hoxa7*.

### 3.2 Hematopoietic Induction of iR9F7-ESCs

**FIGURE 2:**
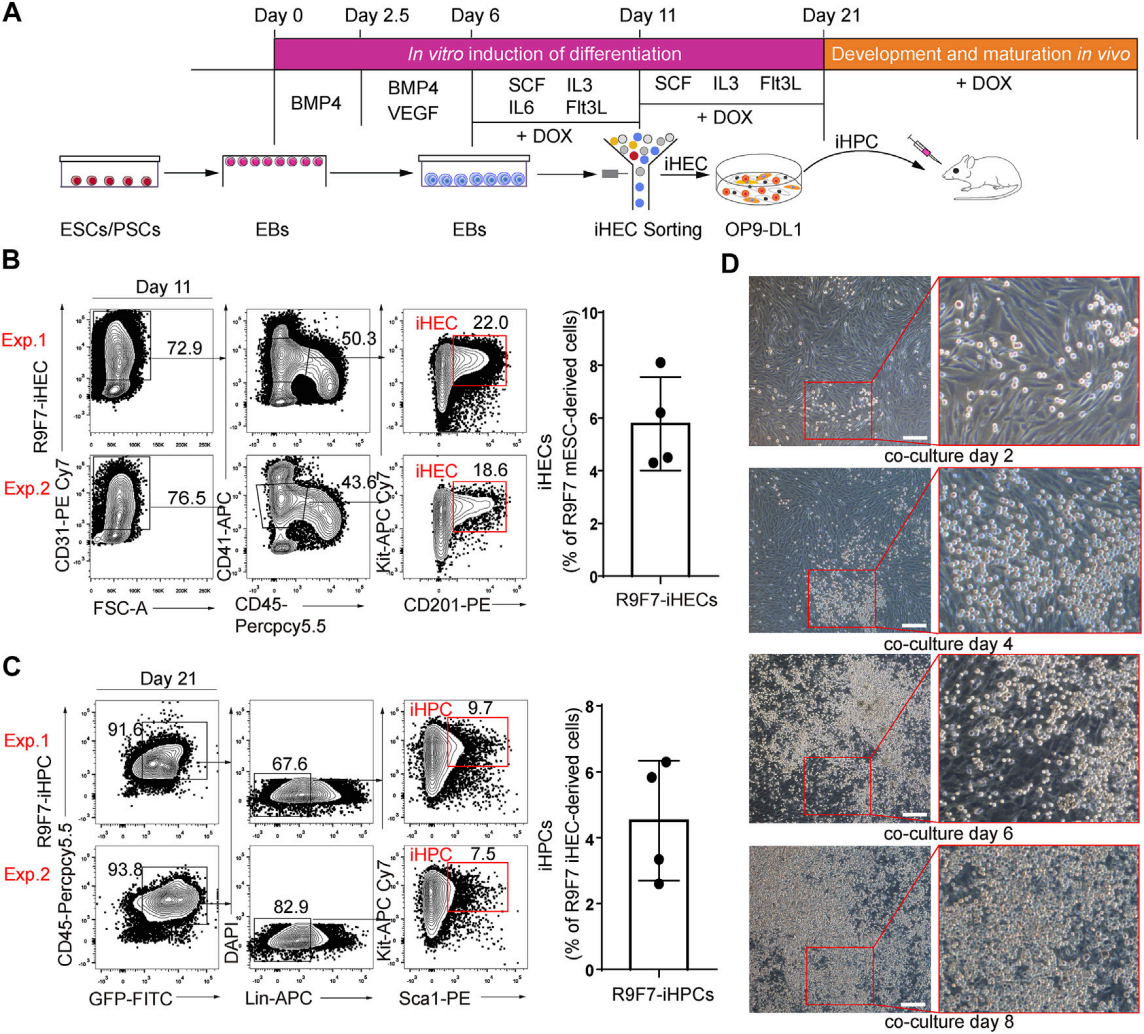
Generation of iHPCs from iR9F7-ESCs *in vitro* **(A)** schematic overview of experimental strategy of hematopoietic induction from ESCs. iHPCs were achieved through stepwise *in vitro* induction and multi-lineage hematopoietic differentiation potential was determined *in vivo* by transplantation **(B)** Representative flow cytometric plots (left) and graph (right) showing the composition of iHECs on day 11 of induction from iR9F7-ESCs. iHEC was defined as CD31^+^CD41^mid^CD45^−^Kit^+^CD201^+^. Two representatives from four independent experiments are shown **(C)** Representative flow cytometric plots (left) and graph (right) showing the composition of iHPCs on day 10 of induction of iHECs on OP9-DL1 stromal cells. iHPC was defined as lin^−^Kit^+^Sca1^+^. Lin was defined as CD2^−^CD3^−^CD4^−^CD8^−^CD11b^−^Gr1^−^Ter119^−^CD19^−^NK1.1^−^TCRγδ^−^. Two representatives from four independent experiments are shown **(D)** Morphology of the sorted iHECs on OP9-DL1 cells on day 2, 4, 6, and 8 of co-culture, respectively. Scale bars, 200 μm.

We used a two-step differentiation protocol (Guo et al., 2020) to induce hematopoietic regeneration from mouse ESCs (Figure 2A). To promote mesoderm differentiation toward hematopoietic cells, embryoid bodies were cultured with BMP4 (days 0–2.5), BMP4 and VEGF (days 2.5–6). Cultured supernatants of AFT024 cell lines with mSCF/mIL3/mIL6/hFlt3L overexpression were used as conditioned medium for the *in vitro* induction of induced hemogenic endothelial cells (iHECs) and subsequent iHPCs. In the presence of doxycycline, embryoid bodies on days 6 to 11 generated iHECs showing an immunophenotype of CD31^+^CD41^mid^CD45^−^Kit^+^CD201^+^ (Figure 2B), which were phenotypically similar to embryonic pre-HSCs (Zhou et al., 2016). To promote the development of iHECs towards iHPCs, embryoid body-derived iHECs were plated on OP9-DL1 stroma, and further educated into Lin^−^Kit^+^Sca1^+^ iHPCs on day 11 to 21 in the presence of doxycycline (Figure 2C). Morphologically, we witnessed the gradually changed cell morphology along with hemogenic and hematopoietic induction (Figure 2D). Thus, iR9F7-ESCs produced iHPCs in dishes.

### 3.3 Engraftment of R9F7-ESC-Derived-iHPCs

**FIGURE 3:**
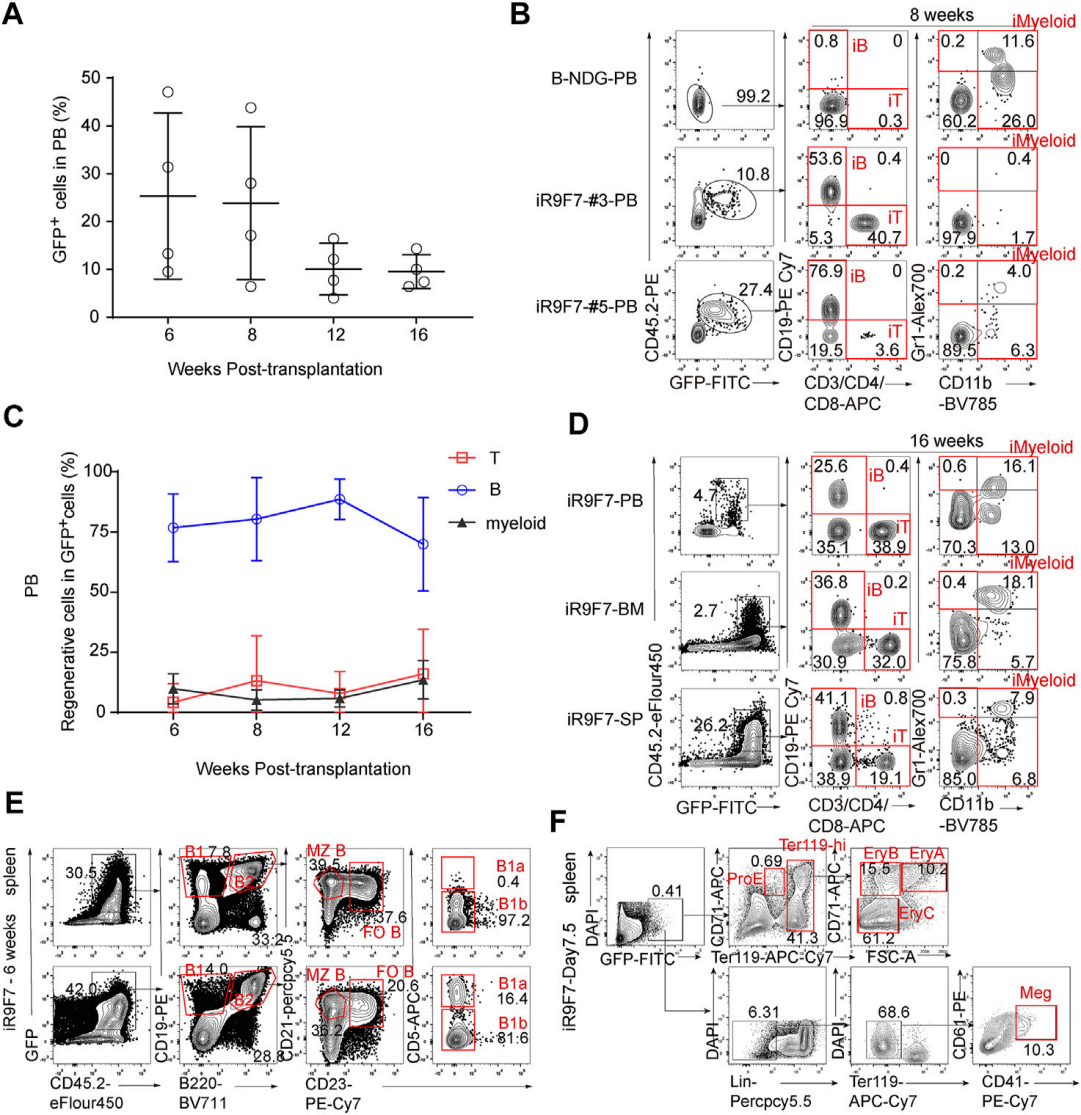
Multi-lineage contribution of R9F7-iHPCs in the recipients **(A)** Frequency of GFP^+^ cells in the peripheral blood (PB) of recipient mice (*n* = 4) at indicated time points post-transplantation. Three million iHEC-derived hematopoietic cells were transplanted into individual B-NDG mouse (CD45.1^+^) irradiated by X-ray (2.25 Gy) **(B)** Representative flow cytometric analysis showing donor reconstitution and lineage composition in the PB of mice transplanted with iHPCs 8 weeks post-transplantation. GFP^+^CD45.2^+^CD19^+^ iB cells, GFP^+^CD45.2^+^CD3/CD4/CD8^+^ iT cells, and GFP^+^CD45.2^+^CD11b^+^ iMyeloid cells detected from two representative recipient mice are shown **(C)** Frequency of regenerated T, B and myeloid cells in the PB of recipients (*n* = 4) at indicated time points post-transplantation **(D)** Representative flow cytometric analysis showing donor reconstitution and lineage composition in the peripheral blood (PB), bone marrow (BM) and spleen (SP) of mice transplanted with iHPCs 16 weeks post-transplantation **(E)** Representative flow cytometric analysis showing donor-derived B cell subtypes in the spleen of mice transplanted with iHPCs 6 weeks post-transplantation **(F)** Representative flow cytometric analysis showing donor-derived erythroid and megakaryocytic populations in the spleen of mice transplanted with iHPCs 7.5 days post-transplantation. Lin was defined as CD2/CD3/CD4/CD8/NK1.1/Gr1/B220.

To further explore the synergistic action of *Runx1*-*Hoxa9*-*Hlf*-*Hoxa7* in engraftment and lineage contribution, 3 million R9F7-ESC-derived-iHPCs on day 21 were injected into sub-lethally irradiated (2.25 Gy) B-NDG mice (8-week-old, CD45.1 strain), Doxycycline (1 mg/ml) was maintained in the drinking water after transplantation. Strikingly, R9F7-iHPCs continuously contributed to multi-lineage reconstitution up to 16 weeks. The frequency of GFP^+^ donor cells in peripheral blood of recipients started at around 25% at 6 weeks post-transplantation, persisted around 20% to week 8 and decreased to 10% at 16 weeks post-transplantation (Figure 3A). At 8 weeks post-transplantation, donor-derived CD19^+^ B cells, CD3/CD4/CD8^+^ T cells, and Gr1/CD11b^+^ myeloid cells were all detected in the peripheral blood of recipient mice (Figure 3B). Of note, the proportion of R9F7-iHPC derived CD19^+^ iB cells were sustained at high level from week 6 (∼75%) to week 16 (∼65%) post-transplantation. In comparison, the proportions of iT cells and iMyeloid cells in the regenerated cell population have been maintained in a relatively low level, with the cell ratio both remaining at 3–10%. This suggests that the R9F7-ESC-derived-iHPCs contributed to myeloid, B, and T cell lineages at various time (Figure 3C). We sacrificed the R9F7-iHPC recipients to check the repopulation in the bone marrow and spleen 16 weeks after transplantation. Flow cytometric analysis showed that there were abundant CD19^+^ iB cells, CD3/CD4/CD8^+^ iT cells, and Gr1/CD11b^+^ iMyeloid cells in these organs in addition to peripheral blood of the recipients (Figure 3D). We also detected different B cell subtypes in the spleen of the recipients 6 weeks post-transplantation (Figure 3E), and the contribution of the iHPCs to erythroid and megakaryocytic lineages in the spleen of the recipients 7.5 days post-transplantation (Figure 3F). Collectively, our data imply that R9F7-overexpressed iHPCs repopulated multi-lineage haematopoiesis in B-NDG mice.

### 3.4 R9F7-iHPCs Efficiently Generated Lineage-Committed Progenitors *in vivo*

**FIGURE 4:**
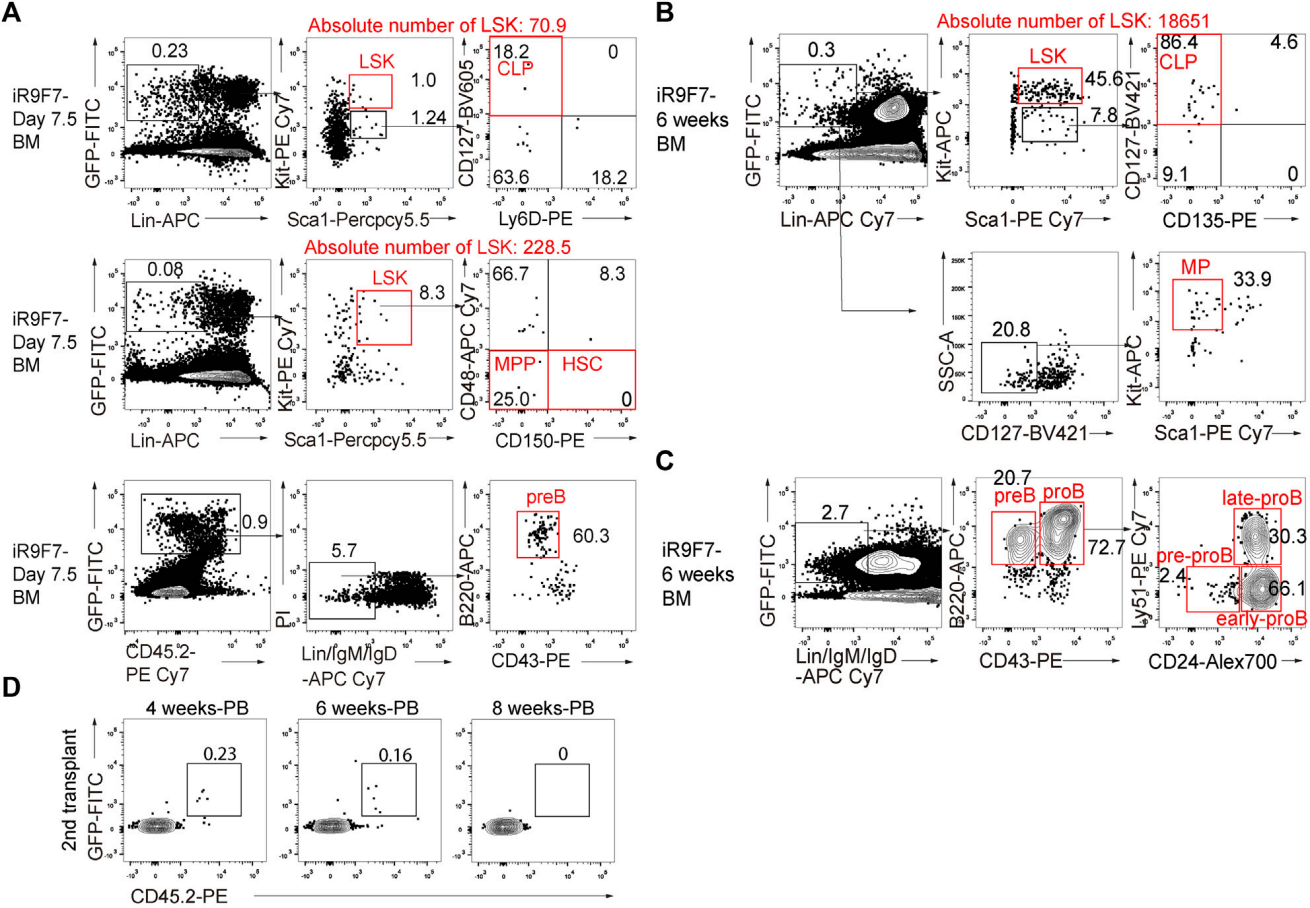
Contribution to lineage-committed progenitors of R9F7-iHPCs in B-NDG recipient mice **(A)** Representative flow cytometric analysis showing donor reconstitution and lineage-committed progenitors from iHPCs in the bone marrow (BM) of recipients 7.5 days post-transplantation. Lin^−^ was defined as CD2^−^CD3^−^CD4^−^CD8^−^CD11b^−^Gr1^−^Ter119^−^B220^−^NK1.1^−^. Common lymphoid progenitors (CLPs) were defined as Lin^−^Kit^mid^Sca1^mid^CD127^+^. Multi-potent progenitors (MPPs) were defined as Lin^−^Kit^+^Sca1^+^CD150^−^CD48^−^. Pre-B cells were defined as Lin^−^IgM^−^IgD^−^B220^+^CD43^−^. Data from one representative recipient mouse are shown **(B)** Flow cytometric analysis of donor-derived immunophenotypic CLPs and myeloid progenitors (MPs) in the BM of recipients 6 weeks after transplantation. MPs were defined as Lin^−^CD127^−^Kit^+^Sca1^−^. Data from one representative recipient mouse are shown **(C)** Flow cytometry analysis of immunophenotypic pro-B cells and pre-B cells in the BM of recipients 6 weeks after transplantation. pro-B (Lin^−^IgM^−^IgD^−^B220^+^CD43^+^) and pre-B (Lin^−^IgM^−^IgD^−^B220^+^CD43^−^) from one representative recipient mouse are shown. Pro-B cells are further divided into three subsets of pre-pro-B, early pro-B and late pro-B on the basis of the expression of CD24 and Ly-51. Lin was defined as Ter119^−^Mac1^−^Gr1^−^NK1.1^−^CD3^−^CD4^−^CD8^−^ **(D)** Flow cytometry analysis of the frequency of R9F7-iHPC-derived cells (GFP^+^CD45.2^+^) in the peripheral blood (PB) of the secondary recipient mice 4, 6, and 8 weeks post-transplantation.

To investigate whether the R9F7-iHPCs can produce lineage-committed progenitors *in vivo*, we investigated the hematopoietic progenitor components in the recipient mice. Firstly, we analysed the bone marrow of the recipients at as early as day 7.5 post-transplantation, and clearly detected donor-derived common lymphoid progenitors (CLPs), multi-potent progenitors (MPPs), and B lymphoid precursors (pre-B) (Figure 4A). We also detected lineage-committed progenitor populations, including CLPs, myeloid progenitors (MPs), and B lymphoid progenitors (pro-B), in the bone marrow of the recipient mice up to 6 weeks after transplantation (Figures 4B,C). We next performed secondary transplantation with donor-derived LSK cells from the primary recipients 6 weeks post-transplantation, but hardly detected chimerism in the peripheral blood of the secondary recipient mice (Figure 4D). Together with the finding showing nearly absence of the immunophenotypic HSCs in the bone marrow of the primary recipients (Figure 4A), we concluded that forced expression of Runx1-Hoxa9-Hlf-Hoxa7 only achieved transient multi-lineage hematopoiesis rather than HSCs regeneration from ESCs.

### 3.5 Genomic Validation of the iR9F7-ESC Origin of Chimeric Cells

**FIGURE 5:**
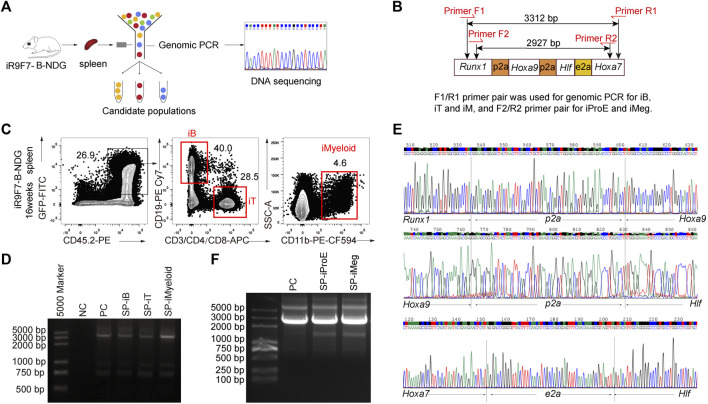
Validation of *Runx1-Hoxa9-Hlf-Hoxa7* insertion in the genome of the regenerated hematopoietic lineages **(A)** Schematic overview of experimental strategy to validate the insertion of *Runx1-Hoxa9-Hlf-Hoxa7* sequence in the genome of the regenerated hematopoietic lineages **(B)** Primer pairs flanking the inserted *Runx1-p2a-Hoxa9-p2a-Hlf-e2a-Hoxa7* sequence element designed for genomic PCR **(C)** Sorting gates of iB, iT, and iMyeloid populations from the spleen of B-NDG recipient mice 16 weeks post-transplantation. The donor-derived iB cells (GFP^+^CD45.2^+^CD19^+^), iT cells (GFP^+^CD45.2^+^CD3^+^), and iMyeloid cells (GFP^+^CD45.2^+^CD11b^+^) were isolated for genome PCR and subsequent sequencing **(D)** PCR bands of *Runx1-Hoxa9-Hlf-Hoxa7* in the regenerated B, T, and myeloid cells from the spleen (SP) of recipients. Water was used as DNA template negative control (NC) to exclude DNA contaminants. The plasmid containing *Runx1-p2a-Hoxa9-p2a-Hlf-e2a-Hoxa7* element was used as positive control (PC). 200 ng DNA of amplified genome of each cell population was used as template for PCR of *Runx1-p2a-Hoxa9-p2a-Hlf-e2a-Hoxa7* element **(E)** Sequencing result of the PCR products of iB cells from one representative recipient mouse visualized by chromas software **(F)** Genome PCR bands of *Runx1-Hoxa9-Hlf-Hoxa7* in the regenerated erythroid progenitor cells (iProE: GFP^+^Ter119^lo^CD71^+^) and megakaryocytes (iMeg: GFP^+^Lin^−^Ter119^-^CD41^+^CD61^+^) from the spleen (SP) of recipients 7.5 days post-transplantation. The plasmid containing *Runx1-p2a-Hoxa9-p2a-Hlf-e2a-Hoxa7* element was used as positive control (PC).

To further verify that the regenerated cells were of iR9F7-ESC origin, genomic PCR assays were performed with donor-derived lineage cells from the iHPC recipients (Figures 5A,B). The GFP^+^CD45.2^+^ B, T, and myeloid cells were isolated from the spleen of the recipients as these cells were abundant in the spleen (Figure 5C). Then, DNA of sorted 2,000 regenerated cell aliquots were extracted, amplified and used for PCR of *Runx1-p2a-Hoxa9-p2a-Hlf-e2a-Hoxa7* sequence, with the plasmid containing the gene tandem co-expression cassette *Runx1*-*p2a*-*Hoxa9*-*p2a*-*Hlf*-*e2a*-*Hoxa7* as the positive control (Figure 5D). Sequencing of PCR products confirmed that the reconstituted lineage cells *in vivo* were derived from iR9F7-ESCs, which carried the inserted *Runx1-p2a-Hoxa9-p2a-Hlf-e2a-Hoxa7* element (Figure 5E). We also validated the iR9F7-ESC-derived cell identity of the GFP^+^ erythroid progenitor cells (GFP^+^Ter119^lo^CD71^+^) and megakaryocytes (GFP^+^Lin^−^Ter119^-^CD41^+^CD61^+^) by genome PCR (Figure 5F). Taken together, these results showed that R9F7-iHPCs repopulate multi-lineage haematopoiesis in B-NDG mice.

### 3.6 Coordination of *Runx1*, *Hoxa9*, *Hlf*, and *Hoxa7* Confers T, B, and Myeloid Cell Lineage Potential on ESC-Derived iHPCs

**FIGURE 6:**
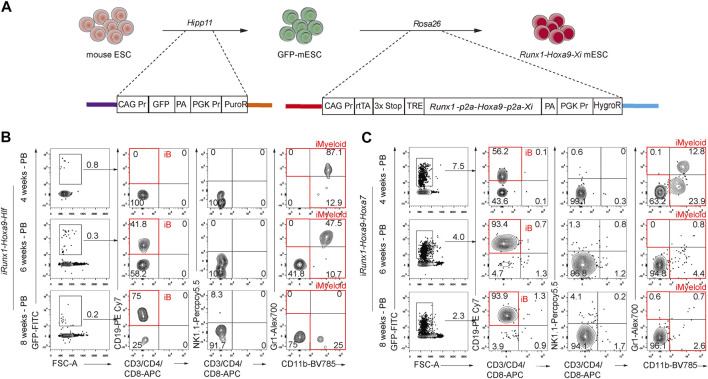
Effects of forced expression of Runx1-Hoxa9-Hlf or Runx1-Hoxa9-Hoxa7 during hematopoietic induction from ESCs **(A)** Schematic diagram of i*Runx1*+*Hoxa9*+Xi mouse ESC lines construction. Xi means transcription factor *Hlf* or *Hoxa7* **(B)** Chimerism and lineage constitution in the peripheral blood (PB) of recipients receiving iHPCs from *iRunx1*+*Hoxa9*+*Hlf* mouse ESCs (iR9F-ESCs) analyzed by flow cytometry 4, 6, and 8 weeks post-transplantation. Data from one representative recipient mouse are shown **(C)** Chimerism and lineage constitution in the PB of recipients receiving iHPCs from *iRunx1*+*Hoxa9*+*Hoxa7* mouse ESCs (iR9A7-ESCs) analyzed by flow cytometry 4, 6, and 8 weeks post-transplantation. Data from one representative recipient mouse are shown.

We wondered whether a combination of three transcription factors would be sufficient to confer multi-lineage engraftment, so we constructed and characterized two inducible cell lines for tandemly expressing *Runx1*-*Hoxa9*-*Hlf* and *Runx1*-*Hoxa9*-*Hoxa7* respectively (Figure 6A). The GFP reporter was inserted into the *Hipp11* locus of mouse ESCs (GFP-ESCs, C57BL/6 background), which allowed tracking of cells with conditional expression of *Runx1*-*Hoxa9*-*Hlf* (R9F) and *Runx1*-*Hoxa9*-*Hoxa7* (R9A7). Following the same two-step method of testing transcription factor combinations (Guo et al., 2020), we achieved *in vitro* regeneration of R9F/R9A7-iHECs and subsequently R9F/R9A7-iHPCs (Supplementary Figure S2). Furthermore, transplantation assays of R9F-ESC-derived iHPCs showed that the R9F-iHPC derivatives could reconstitute only B cell lineage with extremely low contribution (Figure 6B). R9A7 ESC-derived iHPCs somewhat recapitulated the engraftment phenotype of the R9F7-iHPC recipients, despite showing a lower contribution, and the regenerated myeloid cells and T cells could only be temporarily maintained *in vivo* (Figure 6C). Overall, Coordination of *Runx1*, *Hoxa9*, *Hlf*, and *Hoxa7* confers T, B, and myeloid cell lineage potential on ESC-derived iHPCs.

## 4 Discussion

The development and function of HSCs are determined by a complex regulatory gene network. In this study, we established several gene-edited ESC lines and investigated the roles of *Hlf*, *Hoxa7*, *Hoxa9*, and *Runx1* in hematopoietic regeneration from PSCs. Forced expression of these four factors can activate the related intrinsic gene network and drive ESCs towards hematopoietic differentiation to generate iHPCs that lead to multi-lineage hematopoiesis *in vivo*.

With factor-minus-one strategy, *Runx1*-*Hoxa9*-*Hoxa7*-ESC derived iHPCs also achieved multi-lineage hematopoiesis with much lower abundancy. However, *Runx1*-*Hoxa9*-*Hlf*-ESC derived iHPCs only led to B lymphopoiesis with extremely low contribution. *Runx1*-*Hoxa9*-ESC derived iHPCs dominantly possessed T lymphopoiesis ability and generate T lymphopoiesis and *Runx1-Hoxa9-Lhx2*-ESC derived iHPCs lead to complete B lymphopoiesis *in vivo* in our previous studies (Guo et al., 2020; Zhang et al., 2021). Despite *Runx1-Hoxa9-Hlf-Hoxa7*-ESC derived iHPCs achieved transient multi-lineage hematopoiesis, secondary transplantation confirmed the absence of induced HSCs. The above experiments used the same induction protocol and initial ESC line. However, due to the different ectopic expression factors, progenitors with different hematopoietic potentials were induced, but HSCs were still not obtained, indicating that the ideal factors for inducing HSC regeneration *in vitro* are still difficult, and in the future, it might be improved by adopting factors such as *Nupr1* (Wang et al., 2022) and *Hoxa10* (Dong et al., 2020) to promote engraftment and maintenance.

During adult hematopoiesis towards cell maturation, microenvironmental roles become increasingly essential and determine the physiological integrations of newborn terminal blood and immune cells. This is the concern leading us only producing blood seeds in dishes as mimicking hematopoietic maturation niches *in vitro* is far lagging expectation. Based on a two-step strategy, we successfully achieve immune T and B lineage regenerations from PSCs (Guo et al., 2020; Zhang et al., 2021) by two sets of defined factors. In this study, we further achieve transient multi-lineage regeneration from ESCs by four factors based on the same two-step strategy: regenerative seeds *in vitro* and maturation *in vivo*. Despite the absence of induced HSCs, transplantation of iHPCs can regenerate multi-lineage hematopoiesis.

In conclusion, forced expression of exogenous Runx1, Hoxa9, Hlf, and Hoxa7 at hemogenic and hematopoietic stages from PSCs led to generation of the hematopoietic progenitors with the capacity of multi-lineage hematopoietic reconstitution in the immunodeficient recipient mice. The regenerated hematopoietic progenitor cells dominantly contributed to B cells, low proportions of T cells, and myeloid cells *in vivo.* With factor-minus-one strategy, we unveil the combinational roles of Runx1, Hoxa9, Hlf, and Hoxa7 using gene-edited ESC lines, which adds more knowledge and insights on hematopoietic regeneration from PSCs.

## Data Availability

The original contributions presented in the study are included in the article/Supplementary Material, further inquiries can be directed to the corresponding authors.
