# Supplementary material for: Co-Expression of Runx1, Hoxa9, Hlf, and Hoxa7 Confers Multi-Lineage Potential on Hematopoietic Progenitors Derived From Pluripotent Stem Cells
Source: Front Cell Dev Biol. 2022 Apr 28;10:859769. doi: 10.3389/fcell.2022.859769 (PMC9096103; doi:10.3389/fcell.2022.859769)
Supplement: Supplementary file 1 [file DataSheet1.PDF]

Supplementary figures

A

RT-PCR Primers

Gene	Sequence origin	Primers	Amplicon size(bp)	Application
Mouse Gapdh	NM_008085	F: 5'-AGGTCGGTGTGAACGGATTTG-3'	123	RT- PCR
		R: 5'-TGTAGACCATGTAGTTGAGGTCA-3'		RT- PCR
Mouse Runx1	NM_001111023.2	F: 5'-CGTCATGGCAGGCAACGATG-3'	160	RT- PCR
		R: 5'-TGGCGGATTTGTAAAGACGG-3'		RT- PCR
Mouse Hoxa9	NM_152739.4	F: 5'-TGAGAGCGGCGGAGACAAGC-3'	195	RT- PCR
		R: 5'-TCGGTGAGGTTGAGCAGCC-3'		RT- PCR
Mouse Hlf	NM_172563.3	F: 5'-GACAGCTCCCCTTGAACCC-3'	151	RT- PCR
		R: 5'-TGAGAGCGGCGGAGACAAGC -3'		RT- PCR
Mouse Hoxa7	NM_010455.2	F: 5'-TATGTGAACGCGCTTTTTAGCA-3'	166	RT- PCR
		R: 5'-GGGGGCTGTTGACATTGTATAA-3'		RT- PCR

F= forward primer, R= reverse primer.

B

Genomic PCR Primers

Gene	Primers	Amplicon size(bp)	Application
R9F7	F1: 5'-CGATAGATATCtGCTAGCTAGGCCACCATGCGT-3'	3312	Genomic PCR
	R1: 5'-GGCGTCGGCTCCCAGGC-3'		
R9F7	F2: 5'-GGCAACGATGAAAACTACTC-3'	2927	Genomic PCR
	R2: 5'- CTCTGATAGAGGGGGCTGTTGA-3'		

F= forward primer, R= reverse primer.

C

Sequencing Primers

Gene	Primers	Application
Runx1	F1: 5'-CGTATCCCCGTAGATGCCAGC-3'	Sequencing
	F2: 5'-ATCACCTCTTCCTCTGTC-3'	Sequencing
Hoxa9	F1: 5'-TACTATGTGGACTCCTTC-3'	Sequencing
Hlf	R1: 5'-CTGCTGCTCTCATCGTCCA-3'	Sequencing
Hoxa7	R1: 5'-GGGGGCTGTTGACATTGTATAA-3'	Sequencing

F= forward primer, R= reverse primer.

Supplementary Figure 1. Primers and predicted length of PCR products. **(A)** RT-PCR primers. **(B)** Genomic PCR primers. **(C)** Sequencing primers.

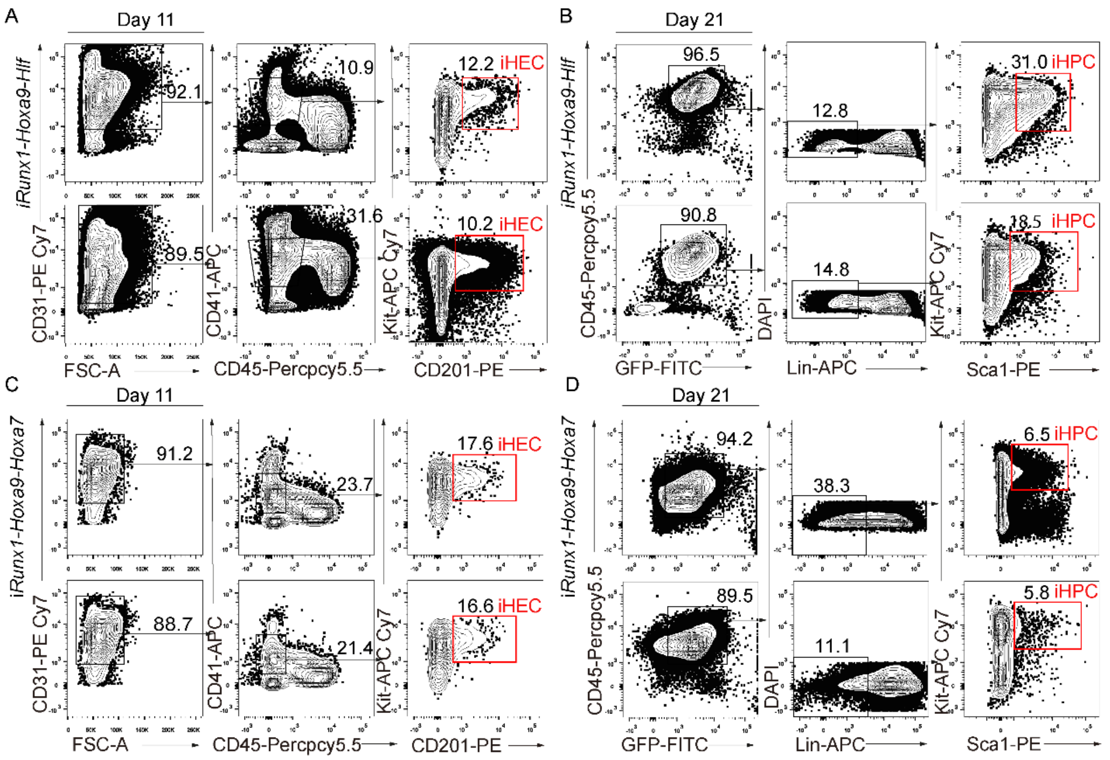

Supplementary Figure 2. iHECs and iHPCs generated from iR9F-ESCs and iR9A7-ESCs *in vitro*. **(A)** Flow cytometric analysis of iHECs on day 11 induction from iR9F-ESCs. iHEC was defined as CD31^{+}CD41^{mid}CD45^{-}Kit^{+}CD201^{+}. Two representatives from five independent experiments are shown. **(B)** Flow cytometric analysis of iHPCs derived from R9F-iHECs. iHPC was defined as Lin^{-}Kit^{+}Sca1^{+}. Lin was defined as CD2^{-}CD3^{-}CD4^{-}CD8^{-}CD11b^{-}Gr1^{-}Ter119^{-}CD19^{-}NK1.1^{-}TCRγδ^{-}. Two representatives are shown. **(C)** Flow cytometric analysis of iHECs on day 11 induction from iR9A7-ESCs. Two representatives from five independent experiments are shown. **(D)** Flow cytometric analysis of iHPCs derived from R9A7-iHECs. Two representatives are shown.
